# Birth weight is associated with obesity and T2DM in adulthood among Chinese women

**DOI:** 10.1186/s12902-022-01194-1

**Published:** 2022-11-18

**Authors:** Pu Song, Hui Hui, Manqing Yang, Peng Lai, Yan Ye, Ying Liu, Xuekui Liu

**Affiliations:** 1 Department of Neurology, Xuzhou Central Hospital, Jiangsu, China; 2 Department of Radiotherapy, Xuzhou Central Hospital, Jiangsu, China; 3 Department of Central Laboratory, Xuzhou Central Hospital, Jiangsu, China; 4 The Graduate School of Xuzhou Medical University, Jiangsu, China; 5 Department of Ultrasonography, Xuzhou Central Hospital, Jiangsu, China; 6 Xuzhou Institute of Medical Science, Xuzhou Institute of Diabetes, Jiangsu, China

**Keywords:** Type 2 diabetes, Birth weight, Obesity

## Abstract

**Background:**

Previous studies have indicated an association between birth weight (BW) and type 2 diabetes mellitus (T2DM), but few studies have explored this relationship under different conditions of obesity in adulthood.

**Methods:**

A total of 4,005 individuals from ten provinces of China were randomly selected to participate in this study. We used a questionnaire to collect age, BW, current weight, height, T2DM history, age at T2DM diagnosis, and other variables. The participants were divided into three groups were according to BW trisection (BW ≤ 2500 g for the lower BW group, 2500 g < BW ≤ 3500 g for the normal BW group, and BW > 3500 g for the higher BW group). The cutoff of overweight and obesity were 25 kg/m^2^ and 28 kg/m^2^, respectively.

**Results:**

The prevalence rates of T2DM among women with lower BW, normal BW and higher BW were 5.2%, 3.6% and 2.0%, respectively. The obesity prevalence rates in the lower BW, normal BW and higher BW groups were 8.1%, 6.7% and 9.0%, respectively. In the obese population, we did not find a relationship between BW and T2DM, but in the nonobese population, we found that with increasing BW, the risk of developing T2DM was reduced. Obese status in adulthood modified the association between BW and the risk of T2DM.

**Conclusion:**

There is a “U” shape association between BW and risk of adulthood obesity in Chinese women, but this trend is not existed between BW and risk of developing T2DM. In non-overweight females, the risk of developing T2DM decreased with increasing BW, but this trend was not observed in overweight females.

**Supplementary Information:**

The online version contains supplementary material available at 10.1186/s12902-022-01194-1.

## Introduction

The COVID-19 pandemic has caused many problems, such as economic stagnation, high hospital admissions, and other societal issues [1]. Many scientific studies that were not relevant to the prevention and control of COVID-19 were suspended to release more medical staff to flatten the curve of the epidemic. Under these conditions, traditional epidemiological studies faced great difficulty because this field requires face-to-face communication between an investigator and a subject. Such face-to-face communication undoubtedly would increase the chance of COVID-19 transmission [2]. However, social networks can be used to conduct epidemiological investigations because they increase the convenience of communication. In the present study, we used online social software to survey the association between type 2 diabetes mellitus (T2DM) in adulthood and birth weight (BW) among Chinese women.

It is well known that the role of intrauterine nutrition during the fetal period is to ensure the healthy growth of the fetus [3]. Malnutrition or overnutrition during pregnancy may cause harm to the fetus [4]. BW is an index that is used to assess the nutrition status of fetuses [5]. The relationship between BW and T2DM has been noted with concern for many years. Barker and his colleagues reported that BW was associated with impaired glucose tolerance in 1998 [6]. QH Xia [7] found that individuals with low BW (< 2500 g) have a higher risk of T2DM (hazard ratio [HR] 1.17) in China. Some systematic reviews reported that lower BW, but not higher BW, can increase the risk of T2DM [8–10] and many academics have indicated that obesity in adulthood may explain the correlation between low BW and T2DM because low BW predisposes individuals to obesity later in life [11]. However, some previous studies found that people with normal BW and higher BW also tend to be obese, and neither positive linear nor J- or U-shaped associations exist between BW and overweight/obesity in adults [12, 13]. Although we hypothesized that these inconsistent conclusions may be caused by differences in ethnicity or the environment later in life, this controversial consequence still needs to be verified in different populations. And not only that, sex differences in metabolic regulation and diabetes susceptibility were concerned in recently years, for example, Tramunt et al. [14] found compared with male, female have higher insulin sensitivity, insulin secretion and incretin responses. However, at present, limited evidence has demonstrated the association between BW and obesity or T2DM in the Chinese population, especially among females.

We collected data via social networks to verify the association between BW and the subsequent risk of obesity and T2DM among the Chinse female population and to develop strategies for preventing these diseases.

## Methods

### Survey method

During the COVID-19 pandemic, traditional epidemiological investigations faced a challenge. The freedom of many people was restricted by the government to stop the spread of the virus. Under this condition, we used social software, such as TikTok and WeChat, to complete our investigation online.

### Survey population and questionnaire

Beginning 2018, we employed social software to spread medical knowledge to the general public online, and the health education videos that we made and released on social software were watched a total of 10 million times. Thousands of people contacted us by social software to receive more medical knowledge. These audiences, who have a certain sense of health and good compliance, were candidate populations for this investigation. We randomly recruited 4,200 female participants into the present study. The inclusion criteria of the study were as follows: 1) the subject was at least 18 years old; 2) the subject could operate the software independently; and 3) the individual knew if she had T2DM. The exclusion criteria were as follows: 1) the subject had severe liver and kidney diseases; 2) the individual had gestational diabetes mellitus; and 3) the subjects could not complete the survey. We designed a questionnaire to survey the participants. The questionnaire included 18 questions, which covered age, BW, current weight, height, T2DM history, age at T2DM diagnosis, hypertension history, age hypertension diagnosis, etc. Self-reported data were the main data source for this study. Height and body weight were measured with participants standing without shoes and heavy outer garments. Body mass index (BMI) was estimated by height and body weight, and the formula as follow: BMI = weight (kg)/height(m^2^).

### Patient and public involvement

The present study is an observed study, and we recruited participants by social software. Before the recruitment, we have announced the research design on the software. We selected 4,200 female individuals from numerous applicants randomly. No one involved in the recruitment to and conduct of the study except for researchers of this study. Results of this survey will be made into health education videos to spread to participants and the general public.

### Quality control

We trained the investigators before the survey to ensure the quality of this study. The training content included how to enroll the interviewee online, how to guide the interviewee to complete the questionnaire online, and how to collect the questionnaire online. This survey lasted for four months, from Dec. 2020 to Jun. 2021, and a total of five investigators collected 4,005 questionnaires. A total of 195 participants did not complete the questionnaire because of lost contact. All the completed questionnaires were input into Excel 2016 for analysis.

### Statistical analyses

We performed survey analysis with SAS 9.3 for Windows (SAS Institute, Cary, NC) to explore the association between BW and T2DM. Age, current height, current weight and BW are shown as the mean ± SD. The frequency of obesity, hypertension and T2DM is shown as the number (ratio%). To observe the clinical characteristics of all the individuals, this sample was divided into three groups according to BW level (T1: BW ≤ 2500 g for the lower BW group, T2: 2500 g < BW ≤ 3500 g for the normal BW group, T3: > 3500 g BW for the higher BW group). The cutoff point of overweight and obesity in these data were 24 kg/m^2^ and 28 kg/m^2^, respectively [15]. ANOVA was performed to analyze the differences in continuous variables, and the LSD test was used to compare the differences between two groups. Logistic regression was carried out to analyze the association between BW and T2DM. Restricted cubic spline was utilized to show the odds ratio (OR) of BW in patients with different BMI levels.

## Results

### The clinical characteristic of all individuals

A total of 4,005 Chinese women were ultimately included in the present study to analyze the association between BW and T2DM. The average age was 48.86 ± 7.99 years old, and the average BMI was 23.38 ± 3.28 kg/m^2^. The average birth weight of the participants was 3063.61 ± 461.91 g. A total of 592 (14.8%) individuals suffered from hypertension, 148 (3.7%) women had T2DM, and 291 (7.3%) participants were obese. The prevalence rates of T2DM among women with lower BW, normal BW and higher BW were 5.2%, 3.6% and 2.0%, respectively (Supplement Fig. 1). Compared with individuals with higher BW, individuals with lower BW had a 3.2% risk of T2DM. The obesity prevalence rates among this study population in the lower BW, normal BW and higher BW groups were 8.1%, 6.7% and 9.0%, respectively (Supplement Fig. 1). Interestingly, subjects with higher BW had a higher obesity rate and a lower prevalence rate of T2DM, and individuals with lower BW have a higher obesity rate and a higher T2DM prevalence rate (Table 1).

**Fig. 1:**
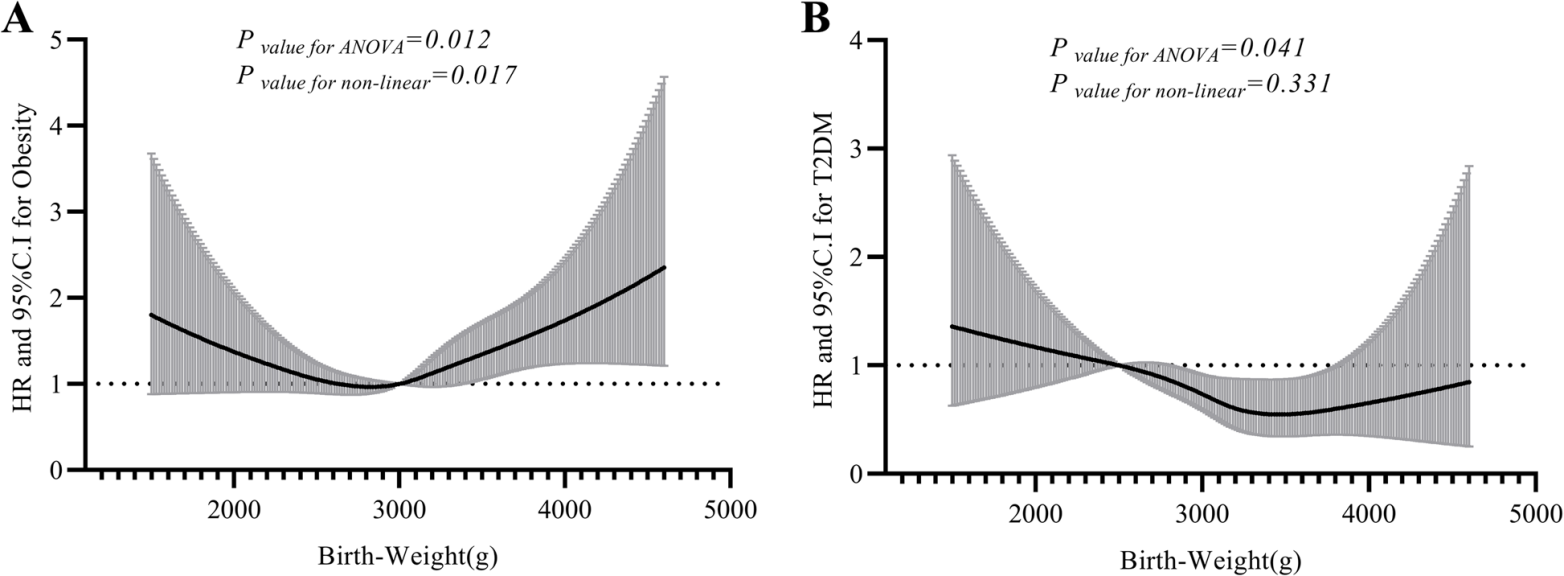
The association between birth weight and obesity (**A**), and the relationship between birth weight and T2DM (**B**) in Chinese women. (HR: hazard ratio, T2DM: Type 2 diabetes mellitus). ANOVA was performed to analyze the risk of obesity or T2DM in different BW levels. Our resluts show that BW is a risk factor of obesity or T2DM in Chinese women. The non-linear test was used to the variation trend of HR. We found there is a linear trend between BW and risk of developing T2DM (**B**), and a non-linear trend existed BW and obesity (**A**)

**Table 1:** Bacsic characteristics of participants according to group of BW (Mean ± SD)

Variables	Birth weight(g)			Total
	Lower BW(< 2500 g)	Median BW(2500 g ~ 3500 g)	Higher BW(> 3500 g)	
n	743	2752	510	4005
Age(years)	49.50 ± 7.55	49.17 ± 7.85	46.29 ± 8.86†	48.86 ± 7.99
Height(cm)	159.83 ± 5.05	161.07 ± 4.79†	162.51 ± 5.43†	161.03 ± 4.98
Weight(Kg)	59.33 ± 9.09	60.69 ± 8.58†	62.18 ± 9.17†	60.63 ± 8.79
BMI	23.22 ± 3.42	23.39 ± 3.19	23.56 ± 3.55	23.38 ± 3.28
Obesity(%)	60(8.1%)	185(6.7%)	46(9.0%)	291(7.3%)
Birth weight (g)	2394.62 ± 313.21	3101.29 ± 195.15†	3832.07 ± 277.27†	3063.24 ± 461.91
Hypertension (%)	123(16.6%)	403(14.6%)	66(12.9%)	592(14.8%)
Type 2 diabetes(%)	39(5.2%)	99(3.6%)	10(2.0%)	148(3.7%)

^†^Compared with the Lower BW group, ^†^*P* < 0.05

### The association between BW and obesity, T2DM in Chinese women using restricted cubic spline analyses

Figure 1A shows that the association between BW and the risk of developing obesity. When BW at the reference point (3000 g), there is a “U” shape existed between BW and obesity. If the participant with BW less than 3000 g, the developing obesity was decreased with increasing BW. On the contrary, If the participant with BW more than 3000 g, the developing obesity was increased with increasing BW (*P* value of ANOVA = 0.012, *P* value of non-linear = 0.017). Figure 1B shows that the relationship between BW and the risk of developing T2DM. The reference point is 2500 g. There is a linear trend between BW and T2DM (*P* value for non-linear = 0.331). The risk of developing T2DM was decreased with increasing BW.

### The association between BW and T2DM in three groups

With the increase in BW, the prevalence rate of T2DM decreased (P for trend = 0.009, Table 2). After adjusting for age, a trend of statistical significance was observed. After adjusting for BMI and hypertension status, the normal BW group was not significantly different from the lower BW group, but the higher BW group was statistically significant from the lower BW group (Table 2).

**Table 2:** The association between BW and T2DM between three groups

Tertiles of BW	Non- T2DM	T2DM	Model1		Model2	
			OR	95%C.I	OR	95%C.I
Lower BW	704(94.8%)	39(5.2%)	1	1	1	1
Median BW	2653(96.4%)	99(3.6%)	0.680	0.465 ~ 0.995	0.687	0.467 ~ 1.011
Higher BW	500(98.0%)	10(2.0%)	0.406	0.200 ~ 0.823	0.387	0.190 ~ 0.792
*P* for trend	0.009		0.006		0.005	

Model1: adjusted Age

Model2: adjusted Age, BMI, Hypertension

### The association between overweight in adulthood and T2DM in different BW groups

Table 3 shows that compared with the non-overweight population, people with overweight in adulthood have a higher risk of developing T2DM at all BW levels. Patients with lower BW combined with overweight had the highest risk of T2DM.

**Table 3:** The association between adulthood overweight and T2DM under different BW levels

		Non-T2DM	T2DM	χ^2^	*P*
Lower BW	Non-overweight	481(96.0%)	20(4.0%)	4.887	0.027
	Overweight	223(92.1%)	19(7.9%)		
Median BW	Non-overweight	1688(97.6%)	42(2.4%)	18.377	< 0.001
	Overweight	965(94.4%)	57(5.6%)		
Higher BW	Non-overweight	310(99.7%)	1(0.3%)	11.141	0.001
	Overweight	190(95.5%)	9(4.5%)		

### Subgroup analysis was used to observe the association between BW and T2DM

In the age subgroups, 35 ~ 45-year-old females exhibited a negative association between BW and T2DM. Females with hypertension also exhibited a negative association. In the overweight population, we did not find a relationship between BW and T2DM, but in the non-overweight population, we found that with increased BW, the risk of developing T2DM decreased (Table 4).

**Table 4:** The logistic regression of BW categories and T2DM in different level of factors

Variables	Beta	Wald	OR	95%C.I	*P*
Age(years)					
< 35	-0.509	2.004	0.601	0.297 ~ 1.216	0.157
≥ 35, < 45	-0.782	8.891	0.457	0.274 ~ 0.765	0.003
≥ 45, < 60	-0.011	0.001	0.989	0.441 ~ 2.221	0.979
≥ 60	-0.215	0.630	0.807	0.475 ~ 1.371	0.807
Hypertension					
No	-0.345	3.151	0.708	0.484 ~ 1.037	0.708
Yes	-0.584	5.323	0.558	0.340 ~ 0.916	0.021
BMI (kg/m^2^)					
< 24	-0.671	8.329	0.511	0.324 ~ 0.806	0.004
≥ 24	-0.268	1.667	0.765	0.510 ~ 1.149	0.197

Note: the model adjusted age, BMI and hypertension, when the factor was stratified, the factor was taken out the model

### Associations between BW and T2DM varied because of obesity status in adulthood

**Fig. 2:**
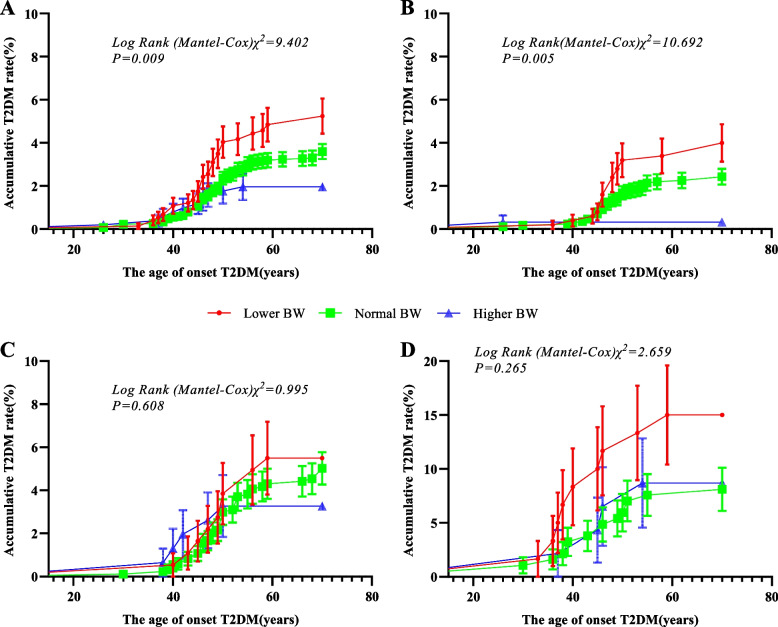
The accumulative incidence rate of T2DM in three groups under the total and different obese status. **A** shows lower BW group has the highest incidence rate of T2DM in three groups, and **B** shows lower BW group has the highest incidence rate of T2DM in three groups under non-overweight or obesity status too. Under the overweight or obesity status, although lower BW group has a higher incidence rate of T2DM, there is no statistical significance

The Fig. 2A shows the accumulative T2DM rate in different BW groups, lower BW group have the highest incidence rate of T2DM in three groups (*P* = 0.009). In normal BMI subgroup, the lower BW participants are also given the highest incidence rate of T2DM (Fig. 2B, *P* = 0.005). In overweight subgroup (Fig. 2C) and obesity subgroup (Fig. 2D), the incidence rate of T2DM were no statistical significance in different BW groups. This result indicated that obese status in adulthood modified the association between BW and the risk of T2DM.

## Discussion

In this study of a sample of Chinese women, we found that there was a significant association between lower BW and an increased risk of T2DM, and BW was associated with the risk of being obesity, with a nonlinear alliance. Subgroup analyses found that the association between BW and T2DM was stronger in the normal BMI adulthood population, and this trend was not observed in individuals who were overweight or obesity in adulthood. Our results not only verified the relationship between BW and T2DM but also found the new argument that being overweight in adulthood can modify the correlation.

Our sample shows that there is a “U” shape between BW and obesity in adulthood, and the higher BW group had the highest prevalence of obesity in three groups. Some previous studies have found that low BW was a risk factor for increased obesity in adulthood. A Chakraborty [16] reported that females with higher socioeconomic status and lower BW had a higher risk of developing obesity (OR = 6.251, 95% CI [1.236 ~ 31.611]). Bischoff [17] found that school-age girls exhibit a positive correlation between BW and fat intake, and fat accumulation in the body leads to obesity. However, a few scholars found no significant relationship between infant nutritional status, which is usually assessed by BW, and metabolic disease. S A Stanner [12] carried out an epidemiological investigation in 1997 and found that the BMI level in adulthood of a person who was born during a famine period was not different from the BMI level in adulthood of people who were born in a food-rich period. The argument that low BW is related to obesity in adulthood is based on the Thrifty Phenotype Hypothesis (TPH) [18–20], which was proposed by Hales and Barker. The TPH supposed that the fetus in utero must adapt to its environment, especially nutritional deficiencies, to ensure brain growth at the expense of other organs, such as skeletal muscle, pancreas, and kidney [21]. Under this condition, metabolic programming has poor access to nutrition, which can lead to metabolic diseases, such as being overweight and T2DM, later in life [22]. However, another argument suggested that there was a difference in BMI-related genes between Asian and European populations [23–25], and diet differences between Eastern and Western populations were one of the causes of the prevalence of metabolic disease [26, 27]. Our results agreed with QH Xia’s report [7], which was conducted in Chinese adults, that a higher BW corresponded with a higher prevalence of obesity and that the population with a lower BW had a lower obesity rate. Wang W reported [24]that three BMI-related genes in Asian populations were not found in European populations, and YP Li reported [28] that there was a stronger association between fetal famine exposure and hyperglycemia in Western countries because Western diets contain more meat, sugary and oils [29], which cause obesity when consumed for a long time. Our results indicated that in the BW group, obesity in adulthood was always a risk factor for developing T2DM.

Many previous studies agreed with the present study, as low BW individuals had a higher prevalence of developing T2DM. In adult Inuit populations in Greenland, a study found that BW was inversely associated with hepatic and peripheral insulin resistance [30]. DH M performed a meta-analysis that selected eight studies and found that BW (< 2500 g) was associated with an increased risk of T2DM (OR, 1.55; 95% CI, 1.39 ~ 1.73) [31]. Although TPH provided a probable relationship between low BW and T2DM, the mechanisms underlying the association are not well understood. Metabolomic profiling between individuals with low BW and T2DM found that subjects with low BW had reduced glycolysis and oxidation ability of postprandial glucose, which may be a possible mechanism [32]. Compared with normal infants, babies with low BW have lower adiponectin levels [33], which may be another reason the incidence rate of T2DM increased. The effect of gene-environment interactions on the development of T2DM cannot be ignored. Cohort studies suggested that genetic susceptibility to obesity and low BW combined with unhealthy lifestyles may synergistically affect the risk of T2DM later in life [34]. Intrinsically, females were less sensitive to insulin resistance than males, so they were at particular risk of developing insulin resistance and therefore more susceptible to the development of T2DM [35]. For this reason, we selected females as our sample population.

The research data of this study were collected by social software online. Compared with traditional epidemiological investigations, the new method of collecting data has some advantages. First, data collected online are not limited to one city or one region. Our sample population was from ten provinces of China. Second, using social software can save time and costs. However, some limitations of this study should be acknowledged. Firstly, because of online collection data, most variables in this study were self-reported. Especially the self-reported variable of T2DM, it is estimated that approximately half of all people with T2DM in China remain undiagnosed [36]. Secondly, this study selected the participants who can operate social software independently may add selection bias to the sample.

## Conclusions

In summary, there is a “U” shape association between BW and risk of adulthood obesity in Chinese women, but this trend is not existed between BW and risk of developing T2DM. In non-overweight females, the risk of developing T2DM decreased with increasing BW, but this trend was not observed in overweight females.

## Supplementary Information

**Additional file 1.** Questionnaire.

**Additional file 2. **Supplement Figure.

## Data Availability

All data generated or analyzed during this study are included in this manuscript.

## Abbreviations

BW: Birth weight
T2DM: Type 2 diabetes mellitus
BMI: Body mass index
OR: Odds ratio
