# Supplementary material for: Birth weight is associated with obesity and T2DM in adulthood among Chinese women
Source: BMC Endocr Disord. 2022 Nov 18;22:285. doi: 10.1186/s12902-022-01194-1 (PMC9673198; doi:10.1186/s12902-022-01194-1)
Supplement: Supplementary file 1 — Additional file 1. Questionnaire. [file 12902_2022_1194_MOESM1_ESM.pdf]

Questionnaire

Guarantee for interviewee

All individual information collected in this survey will be treated as strictly confidential. The record of your name and address will be used only in future follow-up surveys to enable us to contact with you. The computerized data resulting from this survey will not include your name and address. So, nobody will be able to identify any interviewee from the computerized data files. All of the questionnaires will be stored in the locked files containers. This study was reviewed and approved by the ethics committee of the Xuzhou central hospital. The NO. of ethics committee approval is XZXY-LJ-20201110-060.

Interviewee's name: __________________

Current Address: ____

Post Code:

Tel No: __________________ Contact person: _________________________

Tel. No. of Community Office ______ ___________ Person to contact at Community Office__________

Q1: Sex 1. Male 2 Femle

Q2: Current Age _________

Q3: In which province were you born? Province______

Q4: Current height____(cm)

Q5: Current weight____ (kg)

Q6: Do you know your birth weight? 1.yes 2 NO

Q7: What's your birth weight? _______g

Q8: what's your blood pressure currently?________

Q9: How old are you when you have been diagnosised hypertension by doctor?_______

Q10: what's your fasting glucose currently?________

Q11: How old are you when you have been diagnosised T2DM by doctor?______
