# Supplementary figures and images for: Birth weight is associated with obesity and T2DM in adulthood among Chinese women
Source: BMC Endocr Disord. 2022 Nov 18;22:285. doi: 10.1186/s12902-022-01194-1 (PMC9673198; doi:10.1186/s12902-022-01194-1)

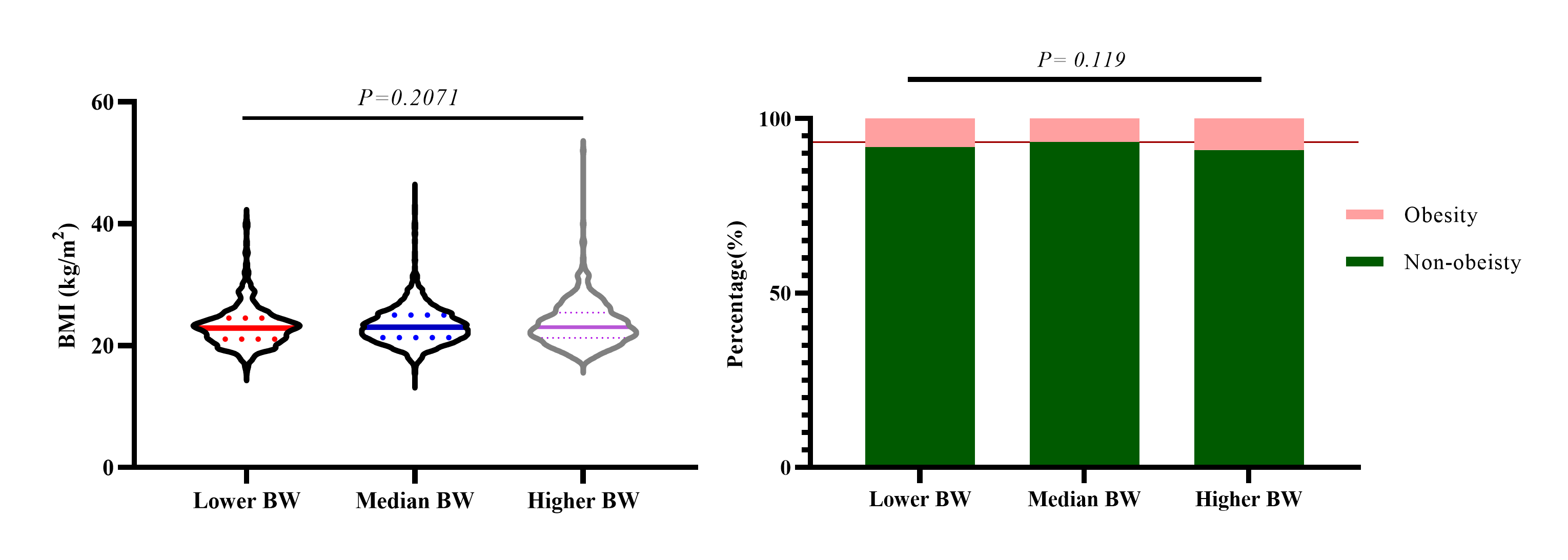

Supplement: Supplementary file 2 — Additional file 2. Supplement Figure. [file 12902_2022_1194_MOESM2_ESM.tif]
